# A behaviour biometrics dataset for user identification and authentication

**DOI:** 10.1016/j.dib.2022.108728

**Published:** 2022-11-07

**Authors:** Nonso Nnamoko, Joseph Barrowclough, Mark Liptrott, Ioannis Korkontzelos

**Affiliations:** Department of Computer Science, Edge Hill University, St Helens Rd, Ormskirk L39 4QP, United Kingdom

**Keywords:** Behaviour biometrics, KMT dataset, Payment authentication, Digital identity, Keyboard and mouse behaviour, Cybersecurity, Identity fraud detection, Machine learning

## Abstract

As e-Commerce continues to shift our shopping preference from the physical to online marketplace, we leave behind digital traces of our personally identifiable details. For example, the merchant keeps record of your name and address; the payment processor stores your transaction details including account or card information, and every website you visit stores other information such as your device address and type. Cybercriminals constantly steal and use some of this information to commit identity fraud, ultimately leading to devastating consequences to the victims; but also, to the card issuers and payment processors with whom the financial liability most often lies. To this end, we recognise that data is generally compromised in this digital age, and personal data such as card number, password, personal identification number and account details can be easily stolen and used by someone else. However, there is a plethora of data relating to a person's behaviour biometrics that are almost impossible to steal, such as the way they type on a keyboard, move the cursor, or whether they normally do so via a mouse, touchpad or trackball. This data, commonly called keystroke, mouse and touchscreen dynamics, can be used to create a unique profile for the legitimate card owner, that can be utilised as an additional layer of user authentication during online card payments. Machine learning is a powerful technique for analysing such data to gain knowledge; and has been widely used successfully in many sectors for profiling e.g., genome classification in molecular biology and genetics where predictions are made for one or more forms of biochemical activity along the genome. Similar techniques are applicable in the financial sector to detect anomaly in user keyboard and mouse behaviour when entering card details online, such that they can be used to distinguish between a legitimate and an illegitimate card owner. In this article, a behaviour biometrics (i.e., keystroke and mouse dynamics) dataset, collected from 88 individuals, is presented. The dataset holds a total of 1760 instances categorised into two classes (i.e., legitimate and illegitimate card owners’ behaviour). The data was collected to facilitate an academic start-up project (called CyberSignature^1^) which received funding from Innovate UK, under the Cyber Security Academic Startup Accelerator Programme. The dataset could be helpful to researchers who apply machine learning to develop applications using keystroke and mouse dynamics e.g., in cybersecurity to prevent identity theft. The dataset, entitled ‘Behaviour Biometrics Dataset’, is freely available on the Mendeley Data repository.

**Specifications Table**

**Table:** 

Subject	Data Science; Computer Science; Engineering and Mathematics
Specific subject area	Specific areas include Artificial Intelligence; Cryptography and Cybersecurity, Applied Machine Learning; Data Engineering; Mathematical Modelling
Type of data	Numeric
How the data were acquired	An application was developed that has a graphical user interface (GUI) similar to a standard online card payment form, including fields for card type, name, card number, card verification code (cvc) and expiry date. Then, user behaviour biometrics commonly known as keystroke, mouse and touchscreen (KMT) dynamics were captured while users entered fictitious card information on the GUI application. To capture such data, the Kivy^2^ Python library was used. The library contains event listeners capable of monitoring any occurrence of events, such as key press, key release, mouse movement, mouse press or mouse release. The GUI application file size is 231 MB and designed to run on laptop or personal computer with the Microsoft Windows operating system installed.
Data format	• Raw KMT data (json format) • Features and Annotation file (json and xlsx format) • Jupyter notebook (IPYNB format)
Description of data collection	1,760 KMT data instances of 2 classes were collected during 88 user sessions on the GUI. Each user session involves 20 iterations of data entry in which the user is assigned a fictitious card information (drawn at random from a pool) to enter 10 times and subsequently presented with 10 additional card information, each to be entered once. The 10 additional card information is drawn from a pool that has been assigned or to be assigned to other users. A KMT data instance is collected during each data entry iteration. Thus, a total of 20 KMT data instances (i.e., 10 legitimate and 10 illegitimate) are collected during each user entry session on the GUI application. All participants were at least 18 years old at the time of data collection.
Data source location	**Institution**: Department of Computer Science, Edge Hill University **City/Town/Region**: St Helens Rd, Ormskirk, Lancashire **Country**: United Kingdom **Postcode**: L39 4QP
Data accessibility	• **Repository name**: Mendeley Data • **Data title**: Behaviour Biometrics Dataset • **Data identification number**: 10.17632/fnf8b85kr6.1 • **Direct URL to data**: https://data.mendeley.com/datasets/fnf8b85kr6

1 Website: www.cybersignature.co.uk; LinkedIn and Twitter: @cybersignature

2 www.github.com/kivy/kivy

## Value of the Data

• The dataset provides a collection of KMT dynamics data related to two classes i.e., legitimate and illegitimate card owner behaviour. Therefore, it enables researchers to apply machine learning methods for user authentication and early detection of fraudulent transactions.
• The dataset can be used for training, testing and validation of other applications whose primary purpose is to develop alternative digital identity and/or frictionless authentication based on user behaviour biometrics.
• The dataset is specifically relevant to technologies that help online payment services conform to the strong customer authentication (SCA) requirement under the second payment services directive (PSD 2) [1].
• The dataset can be used for training, testing and validation of applications whose primary purpose is to secure access and/or provide frictionless second factor authentication, such as corporate password management solutions.
• The dataset can also be used for training, testing and validation of applications whose primary purpose is to develop a blacklist of user behaviour biometrics.

## 1 Data Description

This article presents a dataset containing KMT dynamics of legitimate and illegitimate card owners that could be useful for researchers aiming to apply machine learning techniques to develop digital user identities. The directly applicable task is to distinguish between legitimate card owners and fraudsters during online payment transactions. Traditionally, people enter their card details on payment forms administered on merchants’ websites by payment service companies. Based on a card's first six digits, payment services route the transaction through card schemes (e.g., Visa and Mastercard) to the card issuer who accepts or declines the payment. Cyber-criminals easily hack into one or more of the systems in this network to steal peoples’ card details and use them to commit identity fraud. However, there are hundreds of unique characteristics of a person's behaviour generated whilst entering their card details that are almost impossible to steal, and they can be captured during online payment. These include typing speed, cursor movement habits and preferences to use the mouse, touchpad, or trackball; commonly referred to as KMT dynamics.

**Fig. 1:**
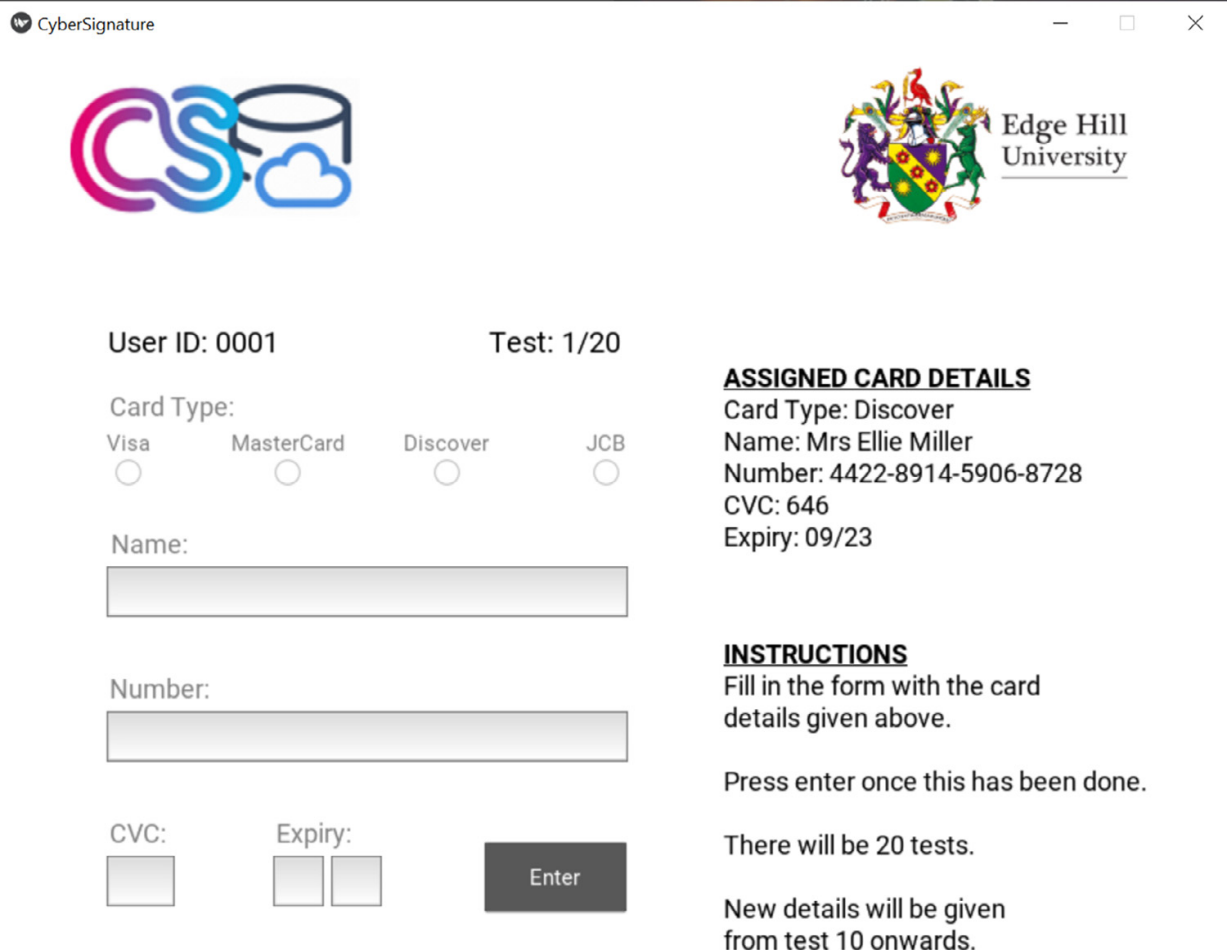
GUI Application for data collection showing a sample set of ‘Assigned Card Details’ with user details and instructions to complete the form.

As part of CyberSignature (www.cybersignature.co.uk/), an academic startup accelerator project, we designed a GUI application that mimics the standard payment form, shown in Fig. 1, and used it to capture user KMT dynamics. Our motivation is to improve online payment security by using KMT dynamics to develop digital user profiles that can be used alongside card details during online payment authentication. Taking advantage of the fact that KMT dynamics can be computationally assessed, we applied machine learning techniques to dynamically process the data such that unique digital profile can be created for legitimate card owners. The profile can then be used during online payment authentication to distinguish between legitimate card owners and cybercriminals.

In this paper, we present an original dataset [2] that contains 1760 instances of raw KMT dynamics data, collected from 88 consenting individuals (male or female aged ≥ 18) whilst they entered fictitious card details on the GUI application shown in Fig. 1. The GUI application file size is 231 MB and designed to run on laptops or personal computers with Microsoft Windows operating system installed. All KMT dynamics data was collected with either a laptop or personal computer, where the GUI application is installed.

**Fig. 2:**
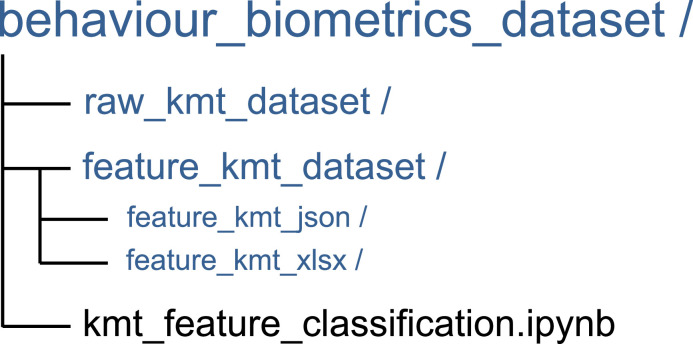
Directory tree of the KMT dataset.

**Fig. 3:**
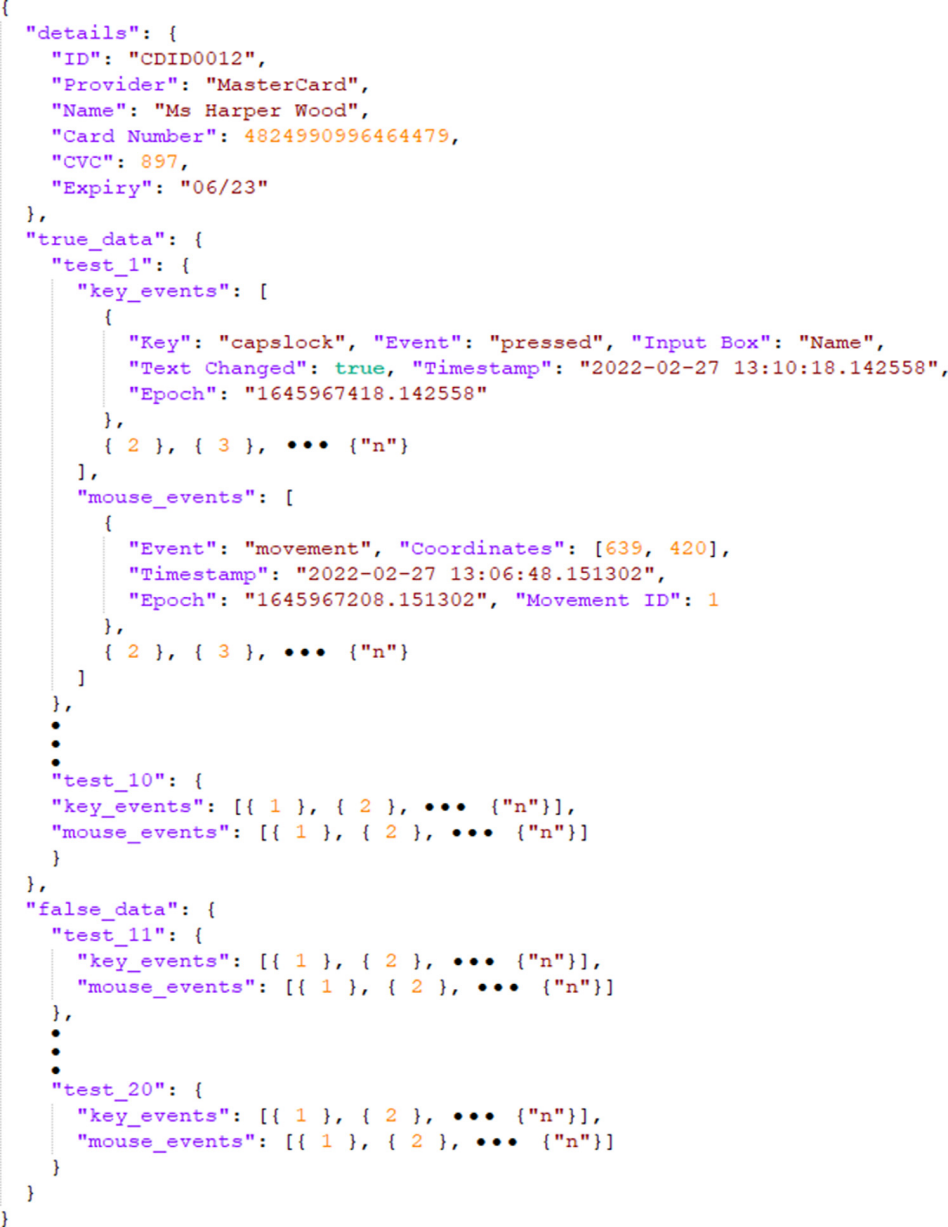
Raw KMT dynamics data structure in json format.
• **‘feature_kmt_dataset’**: contains two sub-folders, namely:
  ○ **‘feature_kmt_json’**: contains 88 json files, each named ‘feature_kmt_user_*n*.json’, where *n* is a number from 0001 to 0088. Each file contains 20 instances of features extracted from the corresponding ‘raw_kmt_user_*n*.json’, including the class labels.
  ○ **‘feature_kmt_xlsx’**: contains 88 xlsx files, each named ‘feature_kmt_user_*n*.xlsx’, where *n* is a number from 0001 to 0088. Each file contains 20 instances of features extracted from the corresponding ‘raw_kmt_user_*n*.json’ file, including the class labels (i.e., ‘1’ for legitimate and ‘0’ for illegitimate).

The raw dataset is stored in json format, in 88 separate files. As shown in Fig. 2, the main folder named ‘behaviour biometrics dataset’ consists of two sub-folders, namely:

• **‘raw_kmt_dataset’**: contains 88 files named ‘raw_kmt_user_*n*.json’, where *n* is a number from 0001 to 0088. Each file contains 20 instances of KMT dynamics data corresponding to a given fictitious card; and the data instances are equally split between legitimate (10 instances) and illegitimate (10 instances) classes. The legitimate class corresponds to KMT dynamics captured from the user that is assigned to the card details; while the illegitimate class corresponds to KMT dynamics data collected from other users entering the same card details. Fig. 3 depicts the data structure within each json file.

In all cases, *n* indicates the user id assigned to the fictitious card detail used to collect the KMT dynamics data.

As shown in Fig. 3 each ‘raw_kmt_user_*n*.json’ file contains nested objects including card ‘details’, and an array of KMT dynamics data collected from legitimate (‘true data’) and illegitimate (‘false data’) users. Specifically, the KMT dynamics data consists of a collection of keyboard and mouse events described in Table 1 and Table 2, respectively.

**Table 1:** A list of captured keyboard events with description.

Keyboard Event	Description
Key	The Kivy ID to identify the actual key pressed e.g. ‘a’ for key A or ‘lshift’ for the left shift key.
Event	Indicates whether the recorded event was a ‘press’ or ‘release’ one
Input Box	The name of the text input box that was in focus on the GUI when the key event was recorded. If there was no input box in focus, it is recorded as ‘Null’
Text Changed	Indicates whether or not the input made a change to any text in the GUI or not. Instances in which text would not be changed represent cases where there is not a text input box in focus, or if the character limit for the text input box has been reached, so that any new inputs do not appear in the input box.
Timestamp	Time stamp of the event in the format: YYYY-MM-DD HH:MM:SS.SSSSSS
Epoch	Denotes the precise number of seconds that have elapsed from the epoch, which in this case, is from January 1, 1970, 00:00:00 (UTC), to the key event.

**Table 2:** A list of captured mouse events with description.

Mouse Event	Description
Event	Denotes whether the recorded event was a ‘movement’, ‘left press’, ’left release’, ‘right press’, ‘right release’, ‘mouse press’, ‘mouse release’, ‘scroll up’, ‘scroll down’
Coordinates	Represents the mouse coordinates on the GUI at the time of the event
Timestamp	Time stamp of the event in the format: YYYY-MM-DD HH:MM:SS.SSSSSS
Epoch	Denotes the precise number of seconds that have elapsed from the epoch, which in this case, is January 1, 1970, 00:00:00 (UTC), to the key event.
Movement ID	For any ’movement’ event, this feature identifies which single mouse movements the event consists of. Each ID is denoted with a single number, and if 0.5 seconds have passed without a new movement, the next movement event would be considered the start of a new mouse movement, and accordingly the ’Movement ID’ value would increment.

As shown in Fig. 2, a folder, ‘feature_kmt_dataset’, with two sub-folders containing files (in json and xlsx formats) is also available in the root directory of the ‘behaviour biometrics dataset’. The files contain minimal features extracted from corresponding ‘raw kmt_user_*n*.json’ files to facilitate the machine learning classification task. Table 3 contains the description of the extracted features with indication of the device and events from which they are deduced. Further details of the feature extraction and classification methods are presented in Sections 2.1 & 2.2, respectively. To allow reproducibility of the results reported in Section 2, we additionally provide a Jupyter notebook file (kmt_feature_classification.ipynb) useful to extract features from the files within the ‘raw_kmt_dataset’ folder and subsequently apply machine learning classification on these features. We also stored the generated features in corresponding files within the ‘feature_kmt_dataset’ folder. Details of the feature extraction process are presented in Section 2.1.

**Table 3:** A list of extracted features with description.

Features	Device	Description
Dwell_avg	keyboard	The average duration that keys on the keyboard are pressed/held. The dwell time for each key press was calculated by finding the ‘press’ key events, and then that keys corresponding ‘release’ event, before calculating the difference in their ‘Epoch’ values.
Flight_avg	keyboard	The average duration between a key release and the next key press. This was calculated by computing the average of all differences in ‘Epoch’ values between a key ‘release’ event and the next key ‘press’ event.
Traj_avg	mouse	The average distance that the mouse travelled within a mouse movement. It was computed by summing all distances travelled from coordinate to coordinate for all the ‘’movement’ events that have the same ‘’Movement ID’, giving a trajectory distance in pixels for each ‘’Movement ID’. The average distance of these ‘’Movement IDs’ was then calculated.

## 2 Experimental Design, Materials and Methods

The dataset enables researchers to apply machine learning methods for user authentication and early detection of fraudulent transactions. In machine learning context, classification aims to find the class to which a new observation belongs. For classification problems, the dataset presented in this paper provides two labelled classes: legitimate (class 1) and illegitimate (class 0). The raw KMT data instances hold between 229 (minimum) and 442 (maximum) keystroke and mouse dynamic events. Thus, it is important to determine which events to use and in what format they can be used to maximise classification performance. This is usually done through a technique commonly known as feature extraction [3].

### 2.1 Feature extraction

In machine learning, feature extraction starts from an initial set of measured data and builds derived values (features) intended to be informative and non-redundant to facilitate the subsequent machine learning and generalization steps [3]. Feature extraction is commonly applied when the input data to a machine learning algorithm is too large and is suspected to contain redundant information. For example, there may be redundancy in the representation of each keyboard and mouse ‘Event’ in Fig. 3, such as ‘pressed’, ‘released’, ‘movement’, ‘left press’, ‘left release’, ‘right press’, ‘right release’, ‘mouse press’, ‘mouse release’, ‘scroll up’, or ‘scroll down’. This data can be transformed into a reduced set of initial features by calculating for example their minimum, maximum and mean occurrence per test iteration. From these initial features, a subset is determined through a technique commonly known as feature selection [4]. The selected features are then expected to contain the relevant information from the input data, so that the desired classification task can be performed by using this reduced representation instead of the complete initial data.

We implemented basic feature extraction by simply calculating the mean occurrence of keyboard dwell and flight time; as well as mean mouse trajectory per KMT dynamics test iteration. Python code (i.e., kmt_feature_classification.ipynb), released with the dataset, performs the following steps:

• Download dataset from Mendeley repository: in this step data is directly downloaded from the Mendeley repository containing the ‘raw_kmt_dataset’ and stored in the current path.
• Import python libraries: this step imports necessary python libraries useful for data manipulation, feature extraction and machine learning classification, such as Numpy, Pandas and scikit-learn.
• Feature extraction and classification: in this step the code applies several calculations on the keyboard and mouse events, to generate the features listed in Table 3; and then applies a machine learning classification algorithm. In the provided code, a data frame containing the relevant features and class labels is generated for each of the ‘raw_kmt_dataset’ files and stored in data frames^3^.
• Visualize data frame generated by feature extraction: this is an optional step that allows the user to visualize a simple data frame of features and labels.

The extracted/selected features are presented with a description in Table 3. We have chosen only a small number of features for simplicity. However, researchers interested in this domain can generate more features from the raw dataset.

### 2.2 Machine learning classification

**Fig. 4:**
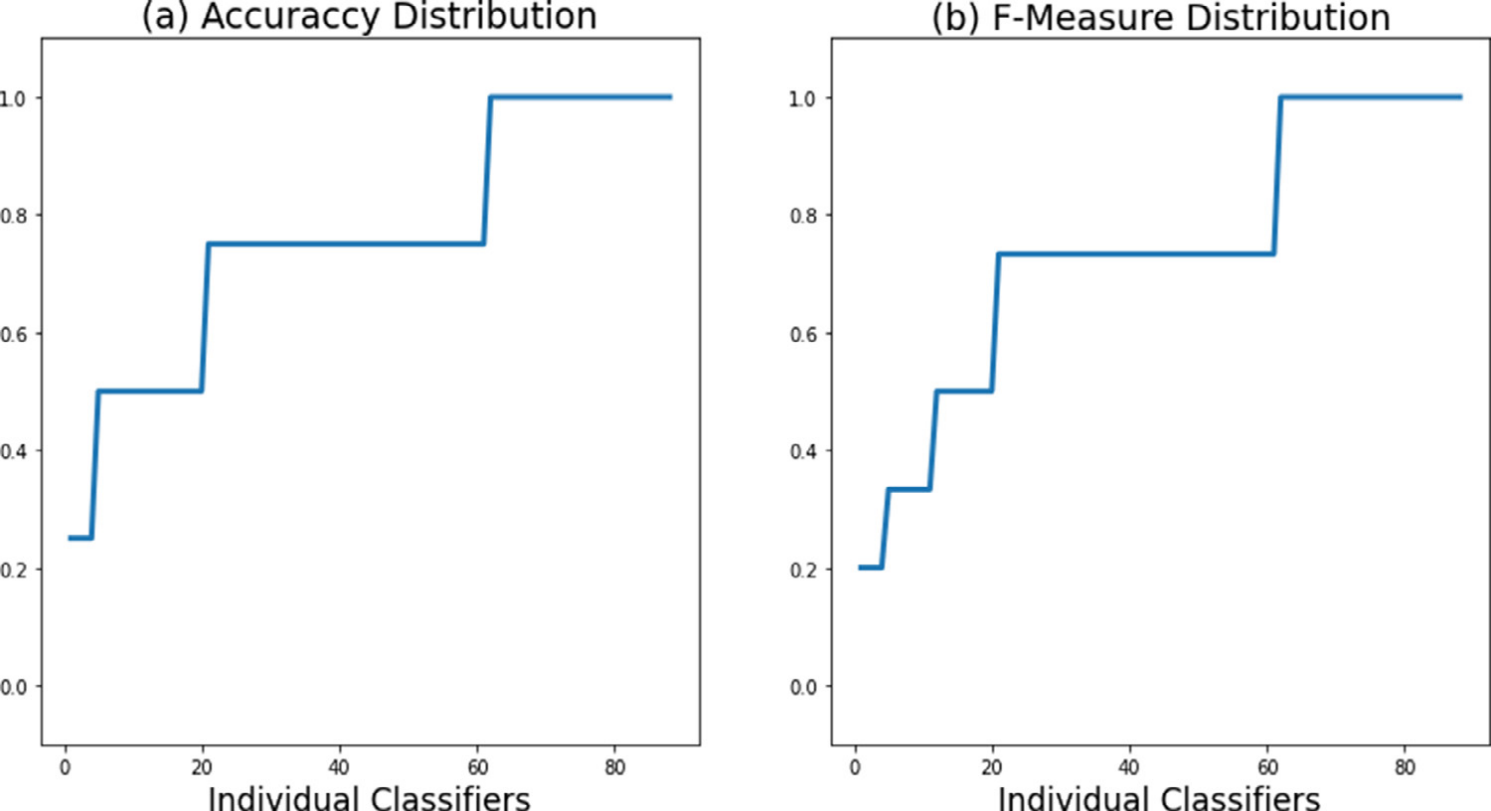
Accuracy and F-Score distribution among the 88 classifiers.

As an example of applying machine learning methods for the classification task, we trained a simple Decision Tree [5] classifier on the proposed dataset. We used python scikit-learn implementation of Decision Tree in its default settings. We considered only the extracted features presented in Table 3 to demonstrate how the dataset can be used for user authentication. The dataset was split in a training and testing part in a stratified manner as follows: 80% train and 20% test. Table 4 shows the minimum and maximum testing accuracy and weighted F-Score obtained from each of the 88 classifiers; as well as the cumulative (mean) values of all the classifiers. The testing accuracy and weighted F-Score distribution among the individual classifiers is also shown in Fig. 4. The individual classifiers are presented in order of performance from minimum to maximum. Detailed description and analysis of the classifier performance falls outside the scope of this paper.

**Table 4:** Accuracy and F-Score results obtained on the classification task.

	Accuracy (%)	Weighted F-Score (%)
Minimum (single classifier)	25.00	20.00
Maximum (single classifier)	100.00	100.00
Mean (88 classifiers)	75.85	73.00

## Ethics Statements

The work involved human subjects entering fictitious data unto a localised GUI application that was designed specifically for the CyberSignature project. No personally identifiable information was collected as part of the data collection. Informed consent was obtained from participants for the publication of data and research and the published data is anonymised. The study was approved by the Science Research Ethics Committee at Edge Hill University under protocol number ETH2122-0336.

## CRediT Author Statement

**N. Nnamoko** and **I. Korkontzelos:** Conceptualization, Methodology, Software, Supervision, Validation; **J. Barrowclough:** Software, Data curation, Visualization, Investigation; **N. Nnamoko**: Writing – original draft preparation, Reviewing & editing; **M. Liptrott, N. Nnamoko** and **I. Korkontzelos**: Writing – review & editing.

## Declaration of Competing Interest

The authors declare that they have no known competing financial interests or personal relationships that could have appeared to influence the work reported in this paper.

## Data Availability

A behaviour biometrics dataset for user identification and authentication (Original data) (Mendeley Data).

## Footnotes

3 Note: A copy of the feature datasets is also available for download from Mendeley repository in both .xlsx and .json formats.
